# Development of Nonlaboratory-Based Risk Prediction Models for Cardiovascular Diseases Using Conventional and Machine Learning Approaches

**DOI:** 10.3390/ijerph182312586

**Published:** 2021-11-29

**Authors:** Mirza Rizwan Sajid, Bader A. Almehmadi, Waqas Sami, Mansour K. Alzahrani, Noryanti Muhammad, Christophe Chesneau, Asif Hanif, Arshad Ali Khan, Ahmad Shahbaz

**Affiliations:** 1 Department of Statistics, University of Gujrat, Gujrat 50700, Pakistan; 2 Department of Internal Medicine, College of Medicine, Majmaah University, Almajmaah 11952, Saudi Arabia; b.almehmadi@mu.edu.sa; 3 Department of Community Medicine and Public Health, College of Medicine, Majmaah University, Almajmaah 11952, Saudi Arabia; w.mahmood@mu.edu.sa; 4 Azra Naheed Medical College, Superior University, Lahore 54000, Pakistan; 5 Department of Family Medicine, College of Medicine, Majmaah University, Almajmaah 11952, Saudi Arabia; m.alzahrani@mu.edu.sa; 6 Centre of Excellence for Data Science and Artificial Intelligence, Universiti Malaysia Pahang, Kuantan 26300, Malaysia; noryanti@ump.edu.my; 7 Centre for Mathematical Sciences, College of Computing and Applied Sciences, Universiti Malaysia Pahang, Kuantan 26300, Malaysia; 8 Department of Mathematics, University of Caen-Normandie, 14032 Caen, France; christophe.chesneau@unicaen.fr; 9 University Institute of Public health, Faculty of Allied Health Sciences, University of Lahore, Lahore 54000, Pakistan; asif.hanif@ahs.uol.edu.pk; 10 Faculty of Computing, Universiti Malaysia Pahang, Pekan 26600, Malaysia; arshadali0343@gmail.com; 11 Department of Cardiac Surgery, Punjab Institute of Cardiology, Lahore 54000, Pakistan; drahmadshahbaz@gmail.com

**Keywords:** nonlaboratory-based features, risk prediction models, machine learning models, LMICs, features importance

## Abstract

Criticism of the implementation of existing risk prediction models (RPMs) for cardiovascular diseases (CVDs) in new populations motivates researchers to develop regional models. The predominant usage of laboratory features in these RPMs is also causing reproducibility issues in low–middle-income countries (LMICs). Further, conventional logistic regression analysis (LRA) does not consider non-linear associations and interaction terms in developing these RPMs, which might oversimplify the phenomenon. This study aims to develop alternative machine learning (ML)-based RPMs that may perform better at predicting CVD status using nonlaboratory features in comparison to conventional RPMs. The data was based on a case–control study conducted at the Punjab Institute of Cardiology, Pakistan. Data from 460 subjects, aged between 30 and 76 years, with (1:1) gender-based matching, was collected. We tested various ML models to identify the best model/models considering LRA as a baseline RPM. An artificial neural network and a linear support vector machine outperformed the conventional RPM in the majority of performance matrices. The predictive accuracies of the best performed ML-based RPMs were between 80.86 and 81.09% and were found to be higher than 79.56% for the baseline RPM. The discriminating capabilities of the ML-based RPMs were also comparable to baseline RPMs. Further, ML-based RPMs identified substantially different orders of features as compared to baseline RPM. This study concludes that nonlaboratory feature-based RPMs can be a good choice for early risk assessment of CVDs in LMICs. ML-based RPMs can identify better order of features as compared to the conventional approach, which subsequently provided models with improved prognostic capabilities.

## 1. Introduction

The surge in cardiovascular diseases (CVDs) and cardiovascular mortality (CVM) has become a real challenge for healthcare systems [1]. However, preventive health policies in high-income countries (HICs) have created a substantial decline in CVDs and CVM in the last two decades [2,3]. This reduction in cause-specific morbidity and mortality reflects the success of preventive health policies, especially the usage of risk prediction models (RPMs) [4]. Predominantly, existing RPMs have been developed, validated and implemented in HICs, although the World Health Organization (WHO) states that almost 80% of CVMs occur in low–middle-income countries (LMICs) [5]. This situation is threatening to LMICs and literature suggests the development of locally customized but methodologically efficient RPMs for CVDs by considering the limitations of existing RPMs.

Usually, RPMs originally established for HIC populations are implemented in LMICs with a slight modification in the mean CVD risk of the model equation called recalibration. However, regression coefficients (βs) of the source RPM are retained in the equation which is originally estimated for the source population (derivation cohort). This trivial modification cannot make this equation truly representative to estimate the risk of CVDs in new populations [4]. Therefore, application of these RPMs in new populations can cause over/underestimation of risk and subsequently misclassification of individuals into low- and high-risk categories. In the literature, there is evidence of the failure of the Framingham heart risk score (a well-established RPM) in the classification of individuals when used in new populations [6,7,8]. Similarly, the QRISK score (another RPM) produced very different CVD risk estimates as compared to the JBS2 score (an RPM) for the same population [9]. Therefore, various researchers suggest that each population has its own RPM to obtain accurate risk estimates, which helps in the reduction in potential disease burden [4,10,11]. Further, existing RPMs are mostly based on laboratory measures which confine their scope and reproducibility to LMICs [12]. The inclusion of laboratory-based measures can improve the predictive accuracy of RPMs. However, it reduces their utility in LMICs due to limited provision of health services and affordability issues. It stimulates researchers to develop local and customized alternative RPMs.

Logistic regression analysis (LRA) is a conventional approach to estimation in RPMs due to its flexible nature of interpretation and less computational complexity. However, LRA assumes linear relationships between explanatory features and logit of the model, which probably oversimplifies the relationships in these disease models [13,14]. This oversimplification can affect the risk estimation process and lead to less-calibrated models. Further, LRA-based existing RPMs do not consider the interaction terms between the explanatory features and assume simple relationships to be estimated. However, in CVD modeling, features are risk factors which are highly associated with each other and it is hard to overlook their interactive effects. The literature has suggested using advanced statistical classifiers which can overcome the limitations of LRA and subsequently increase the predictive performance of the RPMs [5]. Further, the relative importance of features in advanced classifiers can probably be different from the conventional approach. Hence, this study used machine learning (ML) tools to develop and validate the RPMs for prediction of CVD status, considering LRA as a conventional RPM and using nonlaboratory-based features collected from Pakistani settings (which is one of the LMICs).

## 2. Materials and Methods

### 2.1. Study Population and Design

A case–control study was conducted at the Punjab Institute of Cardiology (PIC), Lahore, which is the largest cardiovascular centre in Pakistan. Gender-based matching with a 1:1 ratio was followed to select the subjects from hospital settings. It means male and female cases were matched with male and female controls, respectively. Further, the controls were selected within ±5 years of their corresponding cases. The patients who were registered in the emergency department of the hospital with the first cardiovascular event (except congenital and rheumatic heart diseases (RHD)) and certified by duty physicians were selected as cases for study. Hospital-based matched controls were selected and assessed by duty physicians for any CVD-related signs and symptoms. These controls were the attendants of patients who visited the hospital. This study was approved by the ethical review committee of the PIC hospital and was conducted from September 2018 to February 2019. A total sample of 460, 230 cases and 230 matched controls, were selected. The subjects having an age < 30 years, any history of CVD events, presence of congenital and RHD, or recently changed dietary habits were excluded from the study. Both cases and controls, who followed the above-mentioned criteria in the pre-specified time frame (September 2018 to February 2019), were selected for this study. Informed consent was obtained from all subjects involved in the study. Further details of study design can be seen in our previous works [15,16].

### 2.2. Description and Identification of Important Features

The data frame used in the development of RPMs consists of fifteen features that were derived from the literature [12,17]. These features are purely self-reported information such as age (*f*_1_), gender (*f*_2_), parental history of CVDs (*f*_3_), diabetes mellitus (*f*_4_), hypertension (*f*_5_), smoking history (*f*_6_), physical inactivity (*f*_7_), self-reported general stress (*f*_8_), abdominal obesity (*f*_9_), consumption of high-salt foods (*f*_10_), low fruit consumption (*f*_11_), low vegetable consumption (*f*_12_), high fried foods/trans fats consumption (*f*_13_), red meat/poultry consumption (*f*_14_) and second-hand smoke exposure (*f*_15_). These features were measured on the following scales. Parental history of CVDs (yes = 1, no = 0), diabetes mellitus (present = 1, absent = 0), hypertension (present = 1, absent = 0), smoking history (smoker = 1, never smoker = 0), physical inactivity (moderate to high physical activity = 1 and low profile physical activity = 0), self-reported general stress (not at all to rarely stressful = 0, Sometimes to very stressful = 1) and abdominal obesity (obese = 1 and non-obese = 0) were binary features. Dietary features used following cut-off points, consumption of high-salt foods or snacks ≥1 time a day, deep-fried foods/snacks/fast foods ≥ 3 times a week, low fruit consumption: < 1-time fruit per day, low vegetables consumption: <1-time vegetables daily, red meat and poultry consumption: ≥2 times daily, are treated as yes = 1 otherwise no = 1. Age was originally measured in years and was used in the development of RPMs. However, highly discriminating age groups were derived using the quick, unbiased, efficient, statistical tree (QUEST) algorithm, which is a type of decision tree. These age groups (≤45 years and >45 years) were used when relative feature importance was extracted. The QUEST algorithm is generally used for univariate splits and followed F-statistic for this purpose. It selects the split point for the selected feature and used a stopping criterion for this split process. We used the purity of node as a stopping criterion in determining the discriminating age groups in addition to a significant *p*-value. The F-statistic (F-value = 28.174, d.f.1 = 1, d.f.2 = 458, *p*-value = 0.001) was highest at split point of ≤45 years and >45 years.

**Figure 1:**
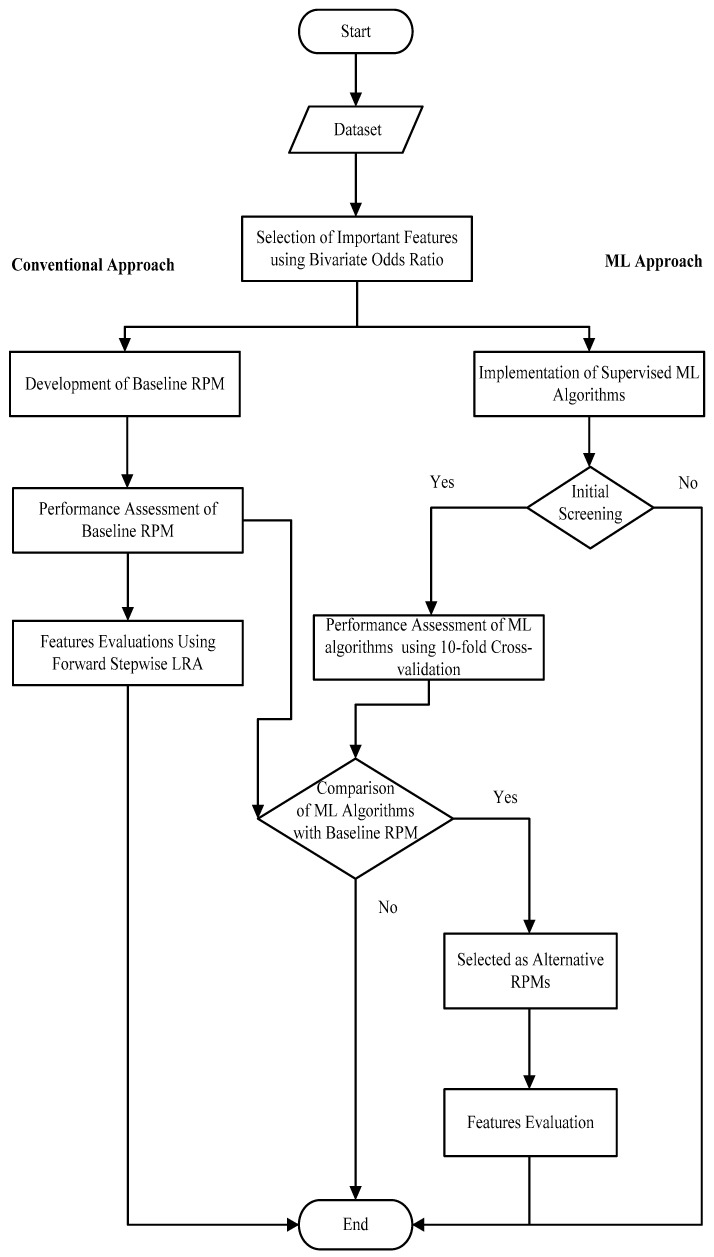
Flowchart for the development of baseline and ML-based RPMs and relative feature importance.

For more precise measurements, an international physical activity questionnaire (IPAQ) was used to assess the status of physical inactivity in the study participants [18]. Abdominal obesity was measured through waist circumference as suggested for the Asian population and used in previous research [19,20]. Dietary features and their cut-off values were the same as used in the literature [12,17]. Before developing the RPMs, the individual association of selected features with CVD status was evaluated through bivariate odds ratio analysis. The associations were tested at a 10% level of significance (α = 10%) and significant features were considered for further process (see Figure 1). It is important to mention here that age and gender are important confounders in such studies. However, these are major risk factors for CVDs too. This study is mainly based on the lifestyle-related risk factors of CVDs. Further, gender-wise disparities in lifestyle-related factors have also been observed in the literature [21]. Therefore, only gender was considered as a confounder, and age was used as an independent risk factor in the study. However, the controls were selected within ±5 years of their cases to avoid the inclusion of very young or very old adults in the study. Possible confounding effects of gender were controlled in two ways; using a matching strategy in the design stage and developing gender-adjusted RPMs at the analysis stage.

### 2.3. Development of Baseline Conventional RPM and Relative Feature Importance

Initially, a baseline RPM was formulated using the conventional approach for CVD prediction as developed in the past [12,17]. Fifteen selected features as discussed in the previous subsection were used and no interaction terms between parameters were considered in the model. This model was formulated using a stepwise regression approach. This approach has further two types; forward selection and backward elimination. The first type starts from the null model and ends at the model with only significant features, while backward elimination starts from a model that contains all features under consideration (full model) and removes the least significant features in each step until a model is finalized with only significant features. However, a forward stepwise selection method was used for estimating the parameters through conventional binary LRA to highlight the significant features (see Figure 1). The overall goodness of fit (GoF) of the model was evaluated through the Hosmer and Lemeshow statistic (*H*) which hypothesizes that the model is fit for prediction (*H*_0_). The significance of Omnibus model coefficients was also observed for the significant contribution of included features in the model. In the end, to make a comparison between baseline RPM and ML models, a confusion matrix was also formed to compute other performance matrices (discussed in the next section). After the performance assessment of the baseline RPM, relative feature importance was also evaluated. In a forward stepwise selection method, features were ranked through the standard method as suggested in the literature [22]. In this method, at every step, −2 *log-likelihood* of the model was computed, which helped in computing the likelihood ratio (LR) test after the inclusion of one variable in the model. The LR test is computed by subtracting the −2 *log-likelihood* of the larger model from the −2 *log-likelihood* of the smaller model. Statistical significance of the LR test (*p* < 0.05) at each step ensured the significance of added variables at the specified step. This stepwise approach was continued until the LR test yielded significant results. The order of the features was observed through the inclusion of each significant feature in the model from the first step to the last step. The features included in the first and last steps would be the most and least discriminating features in the prediction process, respectively. The analysis related to conventional baseline RPM was performed using SPSS version 21.0.

### 2.4. Development of ML-Based RPMs

Three famous supervised ML algorithms were used to train the models for the prediction of CVD status. Artificial neural networks (ANN), support vector machines (SVM) and decision trees (DT), were used to train and test the RPMs. Various configurations of these selected algorithms were implemented to train the models (as presented in Figure 1). As we tried multiple configurations with various combinations of hyperparameters to obtain the most productive ML models, therefore, before the implementation of traditional performance matrices, Cohen’s Kappa-statistic was used as screening criteria. This statistic is used to measure the level of agreement between the observed and the estimated model. It ranges from 0 to 1 and its higher coefficient (≥0.60) reflects the better prediction model. In the initial screening, only those ML models were selected which had a Kappa-statistic (*k*) close to 0.60 or ≥0.60 that showed moderate to a substantial agreement between observed and estimated models. Thresholds of *k* are arbitrary. However, the selection of a threshold depends on the domain of application [23]. The sensitive problems need a higher value of (*k*). In this study, the outcome feature was the first incidence of CVDs which is a group of fatal diseases. Therefore, at least a moderate level of agreement in models was required. Considering this criterion, four ML models—ANN with a single hidden layer, SVM with the linear and radial basis function kernels using sequential minimal optimization (SMO) and random forest (RF)—were finalized with the best-performed combination of hyperparameters. The finalized schemes of these four ML models with their hyperparameters are given as under.

1. ANN with single hidden layer model: The model consisted of a sigmoid activation function with one hidden layer having 8 hidden nodes. The multilayer-perceptron (MLP) with the backpropagation method at 0.3 momentum and learning rate was finalized. Further, weight decay as a regularization technique was also used to avoid over-fitting.
2. The linear kernel SVM model: The SVM was given the entirety of the dataset and mainly trained with two different kernels, which were linear and radial basis function (RBF) kernels. These kernels identified relationships between features within the dataset and tried to find optimal hyperplanes to model the binary outcome. However, linear kernel SVM performed well in the training and testing phases. This SVM model was optimized using the SMO method. Various cost function values (*c_t_*) were implemented to optimize the linear SVM model and *c_t_* = 0.5 provided a good RPM.
3. Radial basis function SVM (RBF-SVM) model: We also tried SVM with an RBF kernel to gauge the possible non-linear patterns within the dataset. The RBF-SVM had two main parameters that needed adjustment: *c_t_* and gamma. Therefore, various cost function values (*c_t_*) were implemented to optimize the model, and *c_t_* and gamma were 1.0 and 0.01, respectively, providing a good RPM.
4. Random forest (RF) model: We have used various types of DT such as C4.5, J48 and RF. However, from this pool of decision trees, the RF performed well and tested further for the development of risk prediction models. RF as an ensemble method was used to create several DT from a set of features selected using the without replacement method. These DT divided the cases and controls into similar subgroups using the most important features. The voting process was used to predict the outcome of the features. A total of 300 DT with a depth of 6 and 3 randomly selected features provided us with a relatively better RPM.

### 2.5. Cross-Validation of ML-Based RPMs and Relative Feature Importance

To develop training and testing samples within the total sample, 10-fold cross-validation was used in the study for ML-based RPMs. Each model was trained ten times by considering each fold as a testing dataset. After the initial screening of ML models through the Kappa-statistic, multiple traditional matrices such as accuracy, root mean square error (RMSE), net reclassification improvement (NRI), sensitivity and specificity were used to evaluate their performance [24,25]. Accuracy is a good choice in a balanced dataset as we had in this 1:1 matched case–control study [26]. Sensitivity and specificity were used to assess the predictive strength of the models in terms of identification of true positive and true negative instances, respectively. In the theory of RPM development, the area under the curve (AUC) and Brier score (BS) are the most preferred measures to assess the discrimination and calibration strength of models, respectively [27]. The value of BS can be calculated through the square of RMSE [28]. Generally, RMSE is used to evaluate the performance of models having continuous outcomes. However, to compute the BS, we have used RMSE in this study. ANN, SVM, and DT-based predictive outputs were evaluated by using these comparative performance matrices, which helped to determine the best RPM/RPMs for future predictions. However, these ML-based models have to face the problem of less interpretability due to their complex black-box nature. Therefore, additional visualization such as partial dependency plots (PDPs) have also been performed to overcome this possible issue. Further, the model which performed better than baseline RPM was used to extract the relative feature importance. In ML-based RPMs, the learner-based approach was followed to determine the relative importance of each feature (see Figure 1). The SVM uses orthogonal vector coordinates orthogonal to the hyperplane to figure out the relative weights of features in the trained model [29]. The input-hidden-output connection weights methodology was used to compute the relative features importance in the ANN model as suggested in the literature [30,31]. The relative feature weights are derived from the Gini index (GI) in RF models [29]. All analyses related to ML models were performed using Weka version 3.8. However, Python 3.9.0 was also used to extract relative feature importance.

## 3. Results

Based on descriptive findings, the average age of the subjects was 48.0 ± 11.31 years. The study sample included 32.2% women in both groups (cases and controls by following the matching strategy). The frequency distribution of all binary features have been presented in Table 1. Consumption of high-salt foods, low fruit consumption, high fried foods consumption, low profile physical activity and smoking are the most prevalent risk features in the studied sample. Further, an association between all binary features and CVDs status was also computed through odds ratio analysis. The bivariate odds ratio analysis found that gender was an insignificant feature with an estimate of 1.00 (0.676–1.479) and a *p*-value = 1.00. However, it was used to formulate the gender-adjusted model. Hypertension was the most significant feature in this bivariate analysis, having an odds ratio of 3.428 (2.163–5.434) and a *p*-value < 0.001. Interestingly, the low vegetable consumption was insignificant at the 5% level of significance. However, at a higher level of significance, i.e., 10%, it was significant (1.383 (0.942–2.029, *p*-value = 0.097)). The findings for other features are; Age in years (1.046 (1.028–1.065, *p*-value = 0.001)), parental history of CVDs (2.292 (1.374–3.824, *p*-value = 0.001)), diabetes mellitus (2.543 (1.633–3.959, *p*-value = 0.001)), smoking history (2.866 (1.890–4.344, *p*-value = 0.001)), physical inactivity (3.030 (2.025–4.52, *p*-value = 0.001)), self-reported general stress (1.921 (1.278–2.889, *p*-value = 0.002)), abdominal obesity (1.768 (1.125–2.778, *p*-value = 0.013)), consumption of high-salt foods (1.843 (1.267–2.680, *p*-value = 0.001)), low fruit consumption, (1.847 (1.237–2.757, *p*-value = 0.003)) high fried foods/trans fats consumption (1.802 (1.233–2.633, *p*-value = 0.002)), red meat/poultry consumption (1.908 (1.080–3.374, *p*-value = 0.025)) and second-hand smoke exposure (1.729 (1.186–2.520, *p*-value = 0.004)). Therefore, all features were considered for further processing.

### 3.1. Baseline Conventional RPM and Relative Feature Importance

A gender-adjusted baseline RPM was formulated. All features used in the baseline RPM were significant (*p* < 0.05) except *second hand smoke exposure* (*p*-value = 0.102). In addition to its insignificance, its inclusion in the multivariate model also caused two more issues. First, its inclusion in the multivariate model caused a severe multicollinearity problem with the main feature of *smoking history*. Second, the *H*-statistic was also significant (*p* > 0.05), reflecting the lack of GoF in the model. Therefore, *second-hand smoke exposure* was dropped from the finalized baseline RPM equation and also from further analysis of the study. The omnibus test for model coefficients was also significant (*χ*^2^ = 235, degree of freedom = 14, *p* < 0.05) which highlighted the significant contribution of included features in the model. *H* statistic was 14.728 (*p* = 0.065) and shows the overall model fitness for prediction. The finalized model equation is provided in the given equation below.

(1) $$\begin{array}{c} Z=-7.69+0.05\left(f_{1}\right)+0.52\left(f_{2}\right)+0.75\left(f_{3}\right)+1.25\left(f_{4}\right)+1.91\left(f_{5}\right)+1.15\left(f_{6}\right)+1.64\left(f_{7}\right)+0.95\left(f_{8}\right)\\ +1.46\left(f_{9}\right)+1.39\left(f_{10}\right)+1.21\left(f_{11}\right)+1.09\left(f_{12}\right)+1.21\left(f_{13}\right)+1.39\left(f_{14}\right) \end{array}$$

Here, Z is the logit and *f*_1_ to *f*_14_ are the features which have been discussed in Section 2.2. The confusion matrix of the saturated model was used to compute the performance matrices of the baseline RPM. The overall accuracy (79.56%) and AUC (0.859) of the model reflect it as an acceptable prediction model for computing heart risk scores. Further, the computed sensitivity, specificity, Kappa-statistic and RMSE through the confusion matrix were 0.804, 0.787, 0.592 and 0.389, respectively (see Table 2). In the end, the LR statistic and −2 *log-likelihood* were used to rank the features. The order of features in the stepwise RPM with their corresponding −2 *log-likelihood* of the model was; physical inactivity (606.06, *p*-value = 0.001), smoking history (570.11, *p*-value = 0.001), hypertension (545.50, *p*-value = 0.001), diabetes mellitus (519.22, *p*-value = 0.001), abdominal obesity (499.10, *p*-value = 0.001), consumption of high-salt foods (476.90, *p*-value = 0.001), low fruit consumption (465.00, *p*-value = 0.001), red meat/poultry consumption (453.90, *p*-value = 0.001), low vegetable consumption (444.21, *p*-value = 0.002), high fried foods/trans fats consumption (433.32, *p*-value = 0.001), age groups (418.94, *p*-value = 0.001), self-reported general stress (406.61, *p*-value = 0.001), parental history of CVDs (402.06, *p*-value = 0.033). 

### 3.2. ML-Based RPMs and Their Performance

Similar to baseline RPM, ML-based RPMs also used the same fourteen features which were used in the finalized baseline RPM as reported in Equation (1). After the initial screening of ML models through the Kappa-statistic, four models were finalized for further processing. The performance assessment of finalized ML models is presented in Table 2. The first finalized model (ANN with 1 hidden layer) provided an RPM with 81.09% accuracy and 0.871 AUC. The sensitivity (0.780) and specificity (0.848) values of the ANN-based model showed consistency in predicting the true positive (TP) and true negative (TN) values of the dataset. The linear SVM reported the best hyperplanes with an accuracy of the model of 80.86%. Further, these two ML models provide 3.7% and 2.7% net reclassification improvement (NRI) than the baseline model. The positive NRI values indicate that these two ML models reclassify the subjects in a more appropriate risk category than the baseline model and improve the classification. The other two models are RBF-SVM and RF. The closeness of RBF-SVM indicates the presence of possible interactions between given features of the dataset. In terms of performance matrices, ANN is the best model among the four selected ML models. The RF model was excellent at yielding sensitivity. However, its other matrices were not better than other ML-based RPMs. The rank order of performance of RPMs was different between performance measures, as given in Table 2, especially in terms of AUC and RMSE. However, it is not compulsory to have improved findings for a particular algorithm on all performance measures, because according to the “No Free Lunch Theorem”, there is no single best optimization algorithm. It implies that there is no single best ML algorithm for predictive modeling problems. Therefore, the best algorithm is that which fulfils the majority of the performance measures. We also prepared five-fold cross-validation results which have been provided as a supplementary table. The pattern of findings was similar in 5- and 10-fold cross-validation.

### 3.3. Performance Comparison between Baseline RPM and ML-Based RPMs

ANN and linear SVM-based RPMs outperform other ML and baseline RPMs in terms of the majority of performance matrices except sensitivity (see Table 2 and Table 3). These two models fulfilled five and six criteria for performance out of six (see Table 3). The RF model showed a slight improvement in RMSE. Overall, ANN and linear SVM-based RPMs outperformed RBF-SVM, RF and baseline RPMs. Therefore, these two better performers proved themselves as good choices for early risk assessment of CVDs as compared to conventional baseline RPM. However, linear SVM-based RPM fulfilled all performance criteria and was found more consistent than ANN and LRA-based RPMs. For more easiness of users, weights assigned by linear SVM can be used for future interpretations and predictions.

### 3.4. Partial Dependency Plots for Identification of Marginal Effects of Features

**Figure 2:**
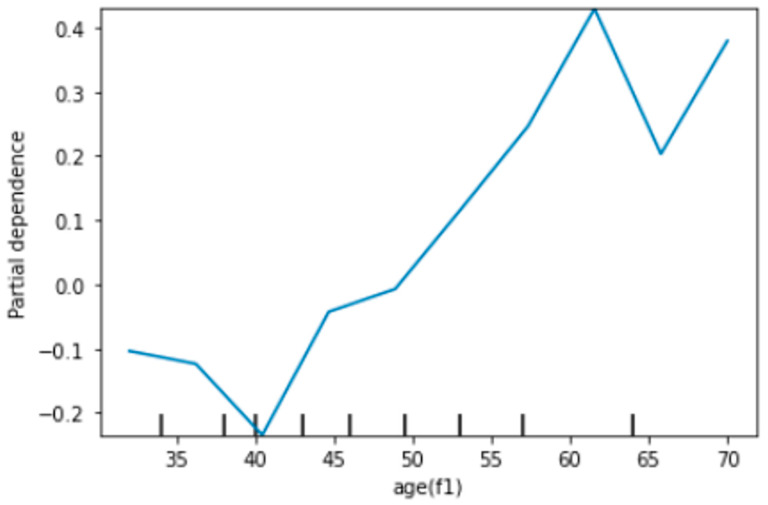
Partial dependence plot (PDP) for visualizing marginal effects of age and CVDs status.

The above-mentioned performance comparison between conventional RPM and ML-based RPMs have shown the advantage of ML-based models in terms of performance. However, unlike the conventional model, the role and nature of the relationship between explanatory features with outcome features are unknown due to the black-box nature of ML-based models. Initially, we developed partial dependency plots (PDPs) to explore the nature of relationships. In this study, except age, all features were binary. Therefore, these features have a linear marginal relationship with the outcome feature. Age was measured at a continuous scale and has depicted its non-linear effects and need to be presented (see Figure 2). The subjects having age under 40 years have shown an inconsistent pattern of risk of CVDs. It is possibly due to multiple types of risk profiles (combinations of risk features) under the age of 40 years. Further, many unexplained biological factors can cause CVDs in this age bracket (30–40 years). However, in higher ages, the prevalence of established classical risk factors is higher that lead to a comprehensible increase in the risk of CVDs.

### 3.5. Relative Feature Importance through Best-Performed ML RPMs

**Figure 3:**
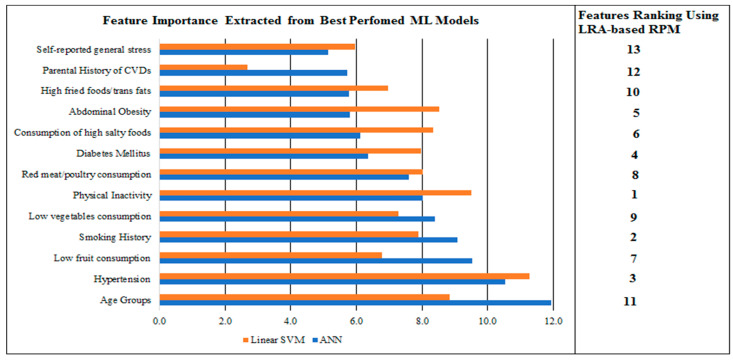
Relative feature importance extracted through best-performed ML and baseline RPMs.

The selected significant thirteen features (excluding gender due to its insignificance) were ranked in order of importance and computed through a learner-based approach as discussed in the methodology section. Here, we presented a horizontal bar chart which is formulated using relative feature importance extracted through best performed ANN and linear SVM-based RPMs (see Figure 3). In the ANN model, age groups, hypertension, low fruit consumption, smoking history and low vegetable consumption were the top five predictive features. On the other hand, linear SVM-based RPM identified hypertension, physical inactivity, age groups, abdominal obesity and consumption of high-salt foods as the top five predictors. The relative feature importance of baseline RPM can also be seen in the same figure.

## 4. Discussion

Accuracy in the prediction of early risk assessment is fundamental in community-centred care. Nonlaboratory features are usually under-appreciated in clinical practice and risk estimation of fatal diseases such as CVDs. This study extends the usage of nonlaboratory-based features (as an alternative to laboratory features in limited resources) to predict CVD events in Pakistani settings. In addition to the conventional baseline RPM, this study provided two alternative but efficient ML-based RPMs which outperformed in the majority of performance matrices. Overall, linear SVM and ANN models were better in overall accuracy, discrimination, and calibration. These ML-based RPMs form the foundations for testing new RPMs in other LMICs which are lacking in their customized models, especially using nonlaboratory features and ML algorithms.

Optimization in risk assessment of CVDs through RPMs is the main dimension of the current study. We found that ML-based RPMs are capable of optimizing the predictive strength of models through the exploration of unobserved patterns and interaction terms in the data sets. In this study, ML-based RPMs expressed their efficiency in predicting the first incidence of CVDs in two ways; improved performance matrices and exploration of new orders of features. These characteristics of ML models augmented our proposition that ML models may perform better as compared to conventional LRA in the estimation of CVD risk. Reflecting on the findings, several observations can be elaborated in the context of published literature. This study found that ML models from the class of ANN and SVM outperformed LRA in performance matrices. The difference between LRA and ML models in terms of predictive strength ranges from 1.30% to 1.53%. The ANN model is specifically used to capture the non-linear and interaction effects of features and provided the highest difference of 1.53%. Indirectly, it indicates the presence of non-linear effects and interaction terms in the selected features which were probably overlooked by the conventional LRA approach [32]. The literature also highlighted that ANN models could improve the prognostic capability of RPMs [33,34]. However, the majority of published literature develops their RPMs using laboratory-based features. RBF-SVM and RF, which are specifically used to gauge the non-linear effects within the dataset, did not outperform the baseline RPM and other ML models. However, the closeness of RBF-SVM and RF performance matrices to linear SVM and baseline LRA models still indicates the presence of slight non-linear effects in the features and CVD status. Despite linear or non-linear relationships between features and CVD status, the better performance of ANN showed its capability to perform equally well in the linear and non-linear dataset. Evidence of the flexible behavior of ANN can be found in the vast body of relevant literature [32,35,36]. The marginal effects of age as highlighted in PDPs has also augmented the presence of non-linear effects in the studied models.

Apparently, the difference in accuracy and discrimination was not substantial between conventional RPM and ML-based RPMs. There could be two main reasons behind this marginal difference in performance. First, the sample size is optimistically adequate for both types of models; LRA- and ML-based RPMs. However, a vast literature is available and reports that the ML models perform better when the sample size is sufficiently large [29,34]. The overall large sample size and proportionally large samples in the training dataset can enhance the capability of ML models to capture the complex (non-linear) patterns of data sets. It subsequently increases the predictive and discriminative strength of ML models. Secondly, there might be chances that the number of features used in this study is not enough for ML models, because ML models perform well in complex data sets in terms of higher dimensionality [37,38]. However, the inclusion of new features such as biomarkers or indicators in disease prediction models needs theoretical knowledge of the domain and adequacy of literature. This limitation restricted the current study to using the same nonlaboratory-based features as used in recent literature for the development of RPMs [12,17]. Irrespective of this limitation, the usage of fewer features in risk prediction is also an advantage and provides strength to current research. The development of parsimonious models is always appreciated in the literature. Therefore, the current study provides models with the good prognostic ability and with minimum nonlaboratory-based features. In the end, in addition to these possible justifications, the literature also reported an apparently marginal difference (1% and above) as a significant contribution to the CVD-related RPMs due to the fatality of this disease [34]. The development of RPMs is purely a classification problem. Therefore, high accuracy is not the only interest for good RPM. In this study, linear SVM and ANN models yielded better discrimination and overall calibration than the baseline RPM.

Most interestingly, we found certain disparities regarding the relative feature importance and their significance from existing RPMs in the literature. This study found that physical inactivity, smoking history, hypertension, diabetes mellitus, and abdominal obesity were the top five predictors of the baseline RPM. In contrast, the *PURE* study concluded that age groups, hypertension, smoking history, diabetes mellitus, and red meat consumption were the top predictors in the RPM [12]. Further, the current study baseline RPM ranked dietary features after these classical features of CVDs and is supported by a recent study that reported that 60% of CVD-related deaths in Pakistan are associated with diet and its patterns [39]. The current study explored a significant positive role of high-salt foods and low vegetable consumption and found consistent with Pakistani literature [40,41]. Surprisingly, the *PURE* study found that usage of salty foods (≥1 time/day) and low vegetable consumption (<1 time/day) were insignificant features in the study, with logistic regression coefficients of −0.16 and −0.24, respectively. However, the similar work of the *INTERHEART* study found that the regression coefficient of these features were 0.12 and 0.21, which indicates their positive role in causing CVDs [17]. In the current study, high fried food consumption was found to have significant importance in baseline RPM, which is aligned with Pakistani literature [42]. In contrast, the existing RPM of the *PURE* study found that high fried food consumption was an insignificant feature in their study. We observed that the current studies, *PURE* and *INTERHEART*, differed in terms of the dietary habits which are associated with regional needs. These differences in findings strengthen the argument for having regional RPMs instead of using a general model to assess CVD risk for all populations.

The argument for developing regional RPMs also got evidence in this study. *PURE* and *INTERHEART* studies-based RPMs included gender-adjusted age groups and found males ≥ 55 years or females ≥ 65 years as a significant age group for CVDs [12,17]. The age categories reflect that males are at more risk of having CVDs at early ages than females. In contrast, multiple Pakistani studies have shown that females have a similar risk of CVDs to their male populations [43,44,45]. Furthermore, since the study dataset was based on a Pakistani sample, and it was quite difficult to find uniform categories of age for scoring purposes in the local context of Pakistan. Therefore, data-driven categories were preferred and found that age > 45 years was significantly associated with CVDs. These newly derived age groups are consistent with local Pakistani studies. For example, a recent cross-sectional study reported that, unlike developed countries, the Pakistani population had a greater risk of CVDs over the age of 40 years [46]. Another study showed that South Asian countries (including Pakistan) reported the onset of CVDs 10 years earlier than other countries [47].

The relative feature importance assigned by the ANN and linear SVM-based RPMs were different from each other. In the ANN, dietary features gained more weight than the classical features of CVDs such as diabetes mellitus and abdominal obesity. In contrast, linear SVM assigned higher weights to classical features such as hypertension, physical inactivity, abdominal obesity and diabetes mellitus, etc. Theoretically, both types of RPMs and their extracted relative feature importance can be explained. More weights to dietary habits and their related features reflect that these features are more pronounced in Pakistani settings and are leading agents for high risk of CVDs. This is consistent with the existing literature [39]. The high prevalence of these features helps in the allocation of more weights in the training process of the ANN algorithm. On contrary, the allocation of higher weights to classical features by linear SVM is due to their established intense effects in causing CVDs. Further, SVM-based models do not require independence of features, which might create a certain combination of features, which leads to better classification and allocation of higher weights to these features. Methodologically, this difference in the allocation of weights in both ML models is simply due to their algorithmic computations and identification of decision boundaries. ANN uses various hyperparameters tuning during the training phase to optimize the performance of the network [37]. ANN uses gradient descent to optimize its parameters. Further, the backpropagation method was used in ANN, which tried to optimize the findings using different weights at certain learning rates and momentum. Furthermore, the regularization technique was also used to avoid over-fitting as discussed in the literature [36]. However, linear SVM searches for linear vectors to separate the classes and generally uses sequential minimal optimization to optimize the findings [29]. In contrast to ANN, linear SVM uses few hyperparameters for its configuration. More importantly, ANN needs a large sample size to train the model [29,48] and SVM can also perform in the range of moderate to large sample sizes [49,50]. Therefore, it is possible to have differences in performance and relative feature importance.

The different role of features in the development of current study RPMs in contrast to existing models would help to strengthen the argument for the need of local RPMs for each population. Further, it would help communities that have low access to laboratory facilities and need intervention for the measurement of risk. In the larger scope, these nonlaboratory-based features can be part of health surveys that are conducted regularly in various countries around the world. These features would help in devising RPMs and act as an initial screening of the population for CVDs and identify potential cases who further confirmed in hospital settings. This screening can lead to need-based community care strategies for the possible reduction in CVDs. Methodologically, this study provides evidence of using advanced data analytics techniques to optimize the risk prediction process.

This study needs to be interpreted within the context of its strengths and limitations. Firstly, in this study, we followed a matched case–control methodology as used in the literature as discussed in followed Section 2. Therefore, we had to adopt the same model development strategy for the baseline RPM which ultimately limited our data analysis techniques and raised possible questions as well. Secondly, the models use baseline data without the follow-up of the participants of the study, which could not capture the effects of time-varying values. The superiority of follow-up designs is not questionable. However, the non-availability of such data sets advocates adopting alternative research designs. Thirdly, there may be additional nonlaboratory features (socio-economic and demographic) that could further improve the performance of RPMs. The inclusion of new features by future researchers can help in the further improvement of ML-based RPMs. Lastly, a large sample size can also be an important factor in improving the findings of ML-based RPMs.

## 5. Conclusions

This study concludes that by using advanced but more flexible ML models, we can optimize the performance of existing models and identify hidden behaviour of features. However, large and multidimensional data sets are recommended for substantial improvement in performance matrices. The different roles and order of features in models of current and existing studies argue that local and customized RPMs should be preferred in the precise estimation of CVD risk. Further, nonlaboratory features are a good alternative for LMICs to develop low-cost RPMs, which can be augmented through the inclusion of background features of participants.

## Figures and Tables

**Table 1:** General characteristics of binary features of the study.

Sr. No	Features	Frequency (%)
1	Gender (*f*_2_)	
	Male	312 (67.8)
	Female	148 (32.2)
2	Parental history of CVDs (*f*_3_)	
	Yes	78 (17.0)
	No	382 (83.0)
3	Diabetes mellitus (*f*_4_)	
	Present	115 (25%)
	Absent	345(75%)
4	Hypertension (*f*_5_)	
	Present	114 (24.8)
	Absent	346 (75.2)
5	Smoking history (*f*_6_)	
	Smoker	142 (30.9)
	Never smoker	318 (69.1)
6	Physical inactivity (*f*_7_)	
	Low profile physical activity	160 (34.8)
	Moderate to high physical activity	300 (65.2)
7	Self-reported general stress (*f*_8_)	
	Sometimes to very stressful	137 (29.8)
	Not at all to rarely stressful	323 (70.2)
8	Abdominal obesity (*f*_9_)	
	Obese	100 (21.7)
	Non-obese	360 (78.3)
9	Consumption of high-salt foods (*f*_10_)	
	Consumption of high-salt foods or snacks ≥ 1 time a day	194 (42.2)
	Consumption of high-salt foods or snacks < 1 time a day	266 (57.8)
10	Low fruit consumption (*f*_11_)	
	<1-time fruit per day	316 (68.7)
	≥1-time fruit per day	144 (31.3)
11	Low vegetable consumption (*f*_12_)	
	<1-time vegetables daily	163 (35.4)
	≥1-time vegetables daily	297 (64.4)
12	High fried foods/trans fats consumption(*f*_13_)	
	Deep-fried foods/snacks/fast foods ≥ 3 times a week	180 (39.1)
	Deep-fried foods/snacks/fast foods < 3 times a week	280 (60.9)
13	Red meat/poultry consumption (*f*_14_)	
	≥2 times daily	58 (12.6)
	<2 times daily	402 (87.4)
14	Second-hand smoke exposure (*f*_15_)	
	More than 1 h of passive smoke exposure per week	226 (49.0)
	Less than 1 h of passive smoke exposure per week	234 (51.0)

**Table 2:** Performance of baseline and ML-based RPMs.

Models	ANN		Linear SVM		RBF-SVM		RF		Baseline RPM	
Confusion Matrix	Case	Control	Case	Control	Case	Control	Case	Control	Case	Control
Case	178	52	186	44	185	45	185	45	185	45
Control	35	195	44	186	54	176	55	175	49	181
Sensitivity	0.780		0.809		0.804		0.804		0.804	
Specificity	0.848		0.809		0.765		0.761		0.787	
Accuracy	81.09		80.86		78.50		78.30		79.56	
AUC	0.871		0.864		0.853		0.856		0.859	
Kappa-statistic	0.622		0.617		0.570		0.565		0.592	
RMSE	0.378		0.382		0.392		0.386		0.389	
NRI	3.7%		2.7%		−2.2%		−2.6%			

**Table 3:** Percentage change in performance matrices of ML-based RPMs to conventional baseline RPM.

Models *	Sensitivity	Specificity	Accuracy	AUC	Kappa-Statistic	RMSE	BS	Number of Criteria Fulfilled
ANN	−2.40%	6.10%	1.53%	1.20%	2.97%	0.378	0.143	5/6
Linear SVM	0.50%	2.20%	1.30%	0.50%	2.50%	0.382	0.146	6/6
RBF-SVM	0.00%	−2.20%	−1.06%	−0.60%	−2.20%	0.392	0.154	0/6
RF	0.00%	−2.60%	−1.26%	−0.30%	−2.68%	0.386	0.149	1/6
						0.389	0.151	

* LRA is a baseline model.

## Data Availability

The source of primary data used in this study is given in the manuscript. However, it is available on request to the corresponding author.

## Supplementary Materials

The following are available online at https://www.mdpi.com/article/10.3390/ijerph182312586/s1, Supplementary Table: Performance of baseline and ML-based RPMs using 5-folds cross-validation.
