# Supplementary material for: Development of Nonlaboratory-Based Risk Prediction Models for Cardiovascular Diseases Using Conventional and Machine Learning Approaches
Source: Int J Environ Res Public Health. 2021 Nov 29;18(23):12586. doi: 10.3390/ijerph182312586 (PMC8657087; doi:10.3390/ijerph182312586)
Supplement: Supplementary file 1 [file ijerph-18-12586-s001.zip › ijerph-1393227-supplementary.pdf]

Supplementary Table. Performance of baseline and ML-based RPMs using 5-folds cross-validation

**Models**	**ANN**		**Linear SVM**		**RBF-SVM**		**RF**		**Baseline RPM**	
Confusion Matrix	Case	Control	Case	Control	Case	Control	Case	Control	Case	Control
Case	178	52	181	49	177	53	180	50	181	49
Control	40	190	41	189	50	180	55	175	50	180
Sensitivity	0.774		0.787		0.770		0.783		0.787	
Specificity	0.826		0.822		0.783		0.761		0.783	
Accuracy	80.00		80.44		77.60		77.20		78.47	
AUC	0.868		0.861		0.844		0.849		0.854	
Kappa-statistic	0.601		0.601		0.560		0.551		0.570	
RMSE	0.381		0.402		0.416		0.412		0.402	
NRI	3.0%		3.9%		-1.7%		-2.6%			
